# Nomogram-Based Risk Model of Small (≤5 mm) Intracranial Aneurysm Rupture in an Eastern Asian Study

**DOI:** 10.3389/fnagi.2022.872315

**Published:** 2022-05-11

**Authors:** Haiyan Lou, Kehui Nie, Jun Yang, Tiesong Zhang, Jincheng Wang, Weijian Fan, Chenjie Gu, Min Yan, Tao Chen, Tingting Zhang, Junxia Min, Renya Zhan, Jianwei Pan

**Affiliations:** ^1^Department of Radiology, School of Medicine, The First Affiliated Hospital, Zhejiang University, Hangzhou, China; ^2^Taimei Medical Technology, Shanghai, China; ^3^Department of Neurosurgery, School of Medicine, The First Affiliated Hospital, Zhejiang University, Hangzhou, China; ^4^Institute of Translational Medicine, School of Medicine, The First Affiliated Hospital, Zhejiang University, Hangzhou, China

**Keywords:** stroke, intracranial aneurysm, rupture, small unruptured aneurysm, nomogram

## Abstract

**Background and Purpose:**

Risk stratification of small unruptured intracranial aneurysms (IAs) (< =5 mm) is important for clinical decision-making and management. The aim of this study was to develop an individualized rupture risk model for small IAs in an eastern Asian population.

**Methods:**

This study retrospectively enrolled 343 patients with ruptured (*n* = 96) and unruptured (*n* = 285) small IAs. Clinical data and aneurysmal morphology were taken into consideration, regression analysis was performed to identify significant variables, and these variables were then incorporated into a predictive nomogram. The diagnostic performance of the nomogram was evaluated using the area under the receiver operating characteristic (ROC) curve (AUC) and calibration plot. Clinical effectiveness was validated by decision curve analysis (DCA). The PHASES score calculated for each case was used for comparison.

**Results:**

The nomogram achieved an AUC of 0.849 (95% *CI*: 0.805–0.893), with a sensitivity of 86.5%, a specificity of 70.9%, and accuracy of 74.7%, which was superior to PHASES score system (AUC = 0.693, sensitivity = 83.6%, specificity = 48.8%, and accuracy = 57.5%). A good agreement between predicted rupture risk and actual rupture status in the small aneurysms was observed, and DCA illustrated the benefit of using the nomogram when decisions needed to be made clinically.

**Conclusions:**

The nomogram based on clinical and morphological risk factors can be useful in assisting clinicians with individualized assessments and benefit-risk balancing in patients with small IAs (< =5 mm).

## Introduction

The prevalence of intracranial aneurysms (IAs) in the Asian population ranges from 2.5 to 3.0%, and the rupture of IAs is the primary cause of aneurysmal subarachnoid hemorrhage (aSAH). Recent studies have shown that most ruptured IAs were smaller than 7 mm (Zheng et al., 2019), and aneurysms smaller than 5 mm were found to be a common cause of aSAH (Dolati et al., 2015).

Since the guidelines for diagnosis and treatment have no specific strategies regarding IAs smaller than 5 mm, clinical decision-making for these patients still remains controversial. Therefore, it is imperative to evaluate the rupture risk and to further improve treatment and management for patients with aneurysms smaller than 5 mm.

Hemodynamic features derived from CTA or DSA were found to be different between ruptured and unruptured aneurysms (Suzuki et al., 2019). However, the acquisition of hemodynamic parameters requires considerable effort in professional manual delineation, and also varies by devices and specialists, in clinical practices. Meanwhile, clinical characteristics and aneurysmal morphology, which are regarded as intuitive and circumstances-unlimited features, have been previously associated with IA rupture (Sonobe, 2010; Ryu et al., 2011; Morita et al., 2012; Kashiwazaki and Kuroda, 2013).

In this 4-year period retrospective observation, we established a clinical decision nomogram-based model using routinely available clinical and morphological parameters for predicting rupture risk of small IAs. To the best of our knowledge, this is the first attempt to develop a decision support system using a nomogram for predicting rupture risk and presenting it as the risk-benefit graph in IAs smaller than 5 mm.

## Materials and Methods

### Patients and Clinical Data

In this study, patients diagnosed with small IAs between January 2016 and December 2019 were retrospectively included. The inclusion criteria were as follows: patients diagnosed with aneurysms by computed tomography angiography (CTA) or three-dimensional digital subtraction angiography (DSA) and patients with aSAH identified on conventional computed tomography (CT) scan; accessible clinical and radiological data. The exclusion criteria were absence of clinical data or high-quality radiological data; age <18 years old; history of other central nervous system diseases; craniocerebral trauma and craniotomy; and other systemic diseases, such as severe hepatic and renal dysfunction. Clinical characteristics included age, sex, INR, history of alcohol/smoke intake, systolic and diastolic blood pressure (BP) at admission, history of antiplatelet and anticoagulant used, family history of aneurysmal SAH; and pre-existing comorbidities (hypertension, diabetes mellitus, hyperlipidemia, cerebro-cardiovascular complications, such as cerebral infarction and myocardial infarction).

### Morphological Parameters of Aneurysms

Morphological characteristics included aneurysmal multiplicity, presence of daughter sac, sidewall or bifurcation type, location and aneurysm max diameter, aneurysm height, aneurysm width and neck width, aspect ratio (AR), bottle-neck ratio (BNR), and height-width ratio (HWR). The definition of max diameter was the largest distance within the aneurysm sac from cross-sectional projection. Aneurysm height was defined as the maximum distance of the dome from the neck center, and aneurysm width represented for the maximum distance of dome perpendicular to aneurysm height. Neck width indicated the width of the aneurysm in the neck plane, from where the aneurysmal sac pouched outward from the parent vessel. AR, BNR, and HWR were calculated by the ratio of aneurysm height to neck width, aneurysm width to neck width, and aneurysm height to aneurysm width, respectively. Morphological parameters were measured on DSA or CTA images and were calculated in the same way as in Ref. (Jiang et al., 2015).

### PHASES Score Assessment

The PHASES risk score (ranges from 0 to 22) was developed in a study for predicting the rupture risk of IAs in the population of Finland, Japan, and Netherlands, and is now generally used for patient management (Greving et al., 2014). The score considers six indicators (population, hypertension, age, size of aneurysm, earlier SAH from another aneurysm, and site of aneurysm), and the points associated with each indicator can be added up to obtain the total risk score. All abovementioned morphological parameters and the PHASES score for each case were assessed by a neurosurgeon with 7 years of experience.

### Nomogram-Risk Model Development and Validation

A nomogram was modeled based on the selected variables from the results of multivariable logistic regression analysis. The discriminative performance of the model was measured by the receiver operating characteristic (ROC) and the corresponding area under the curve (AUC). The fitness of the model was assessed by the Hosmer-Lemeshow test (*p* > 0.05 was regarded as a good fit), and the calibration plot graphically illustrated the agreement between predicted rupture probabilities and observed rupture. Additionally, the net benefit (a value of 0 indicates no benefit and higher values indicate more benefit) of the model was quantified, and thus clinical utility was demonstrated by decision curve analysis (DCA) (Vickers and Elkin, 2006). The model was validated using bootstrapped resampling of 200. Model development and validation were performed using R Studio (version 1.3, Boston, MA, USA).

### Statistical Methods

Continuous variables were summarized asmean ± SD ormedian with interquartile ranges (IQRs), and categorical variables were reported as percentage. A Mann–Whitney *U*-test or *t*-test was applied to continuous variables and Fisher's exact test to categorical variables to investigate the association with rupture. The covariates with *p* <0.2 in univariate analysis were considered formultivariable logistic regression analysis. Significant factors (*p* < 0.05 with 2-tailed) in the multivariable analysis were identified as predictors of rupture events. The univariate and multivariable analyses were performed with SPSS 15.0 (IBM, Chicago, IL). The performance of the nomogram-risk model was compared to PHASES score in AUC value, sensitivity, specificity and accuracy.

## Results

**Figure 1:**
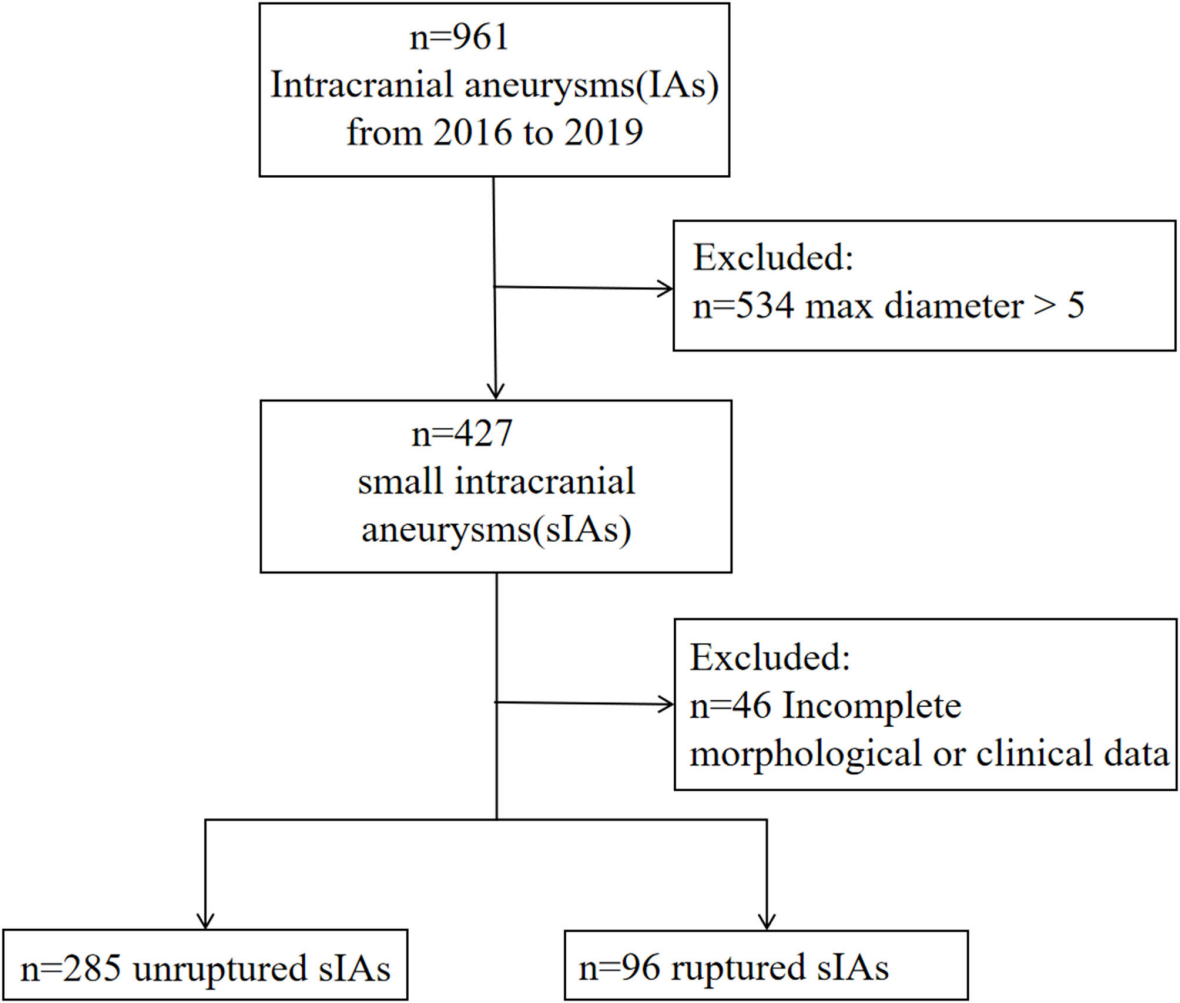
Flowchart of this study.

**Figure 2:**
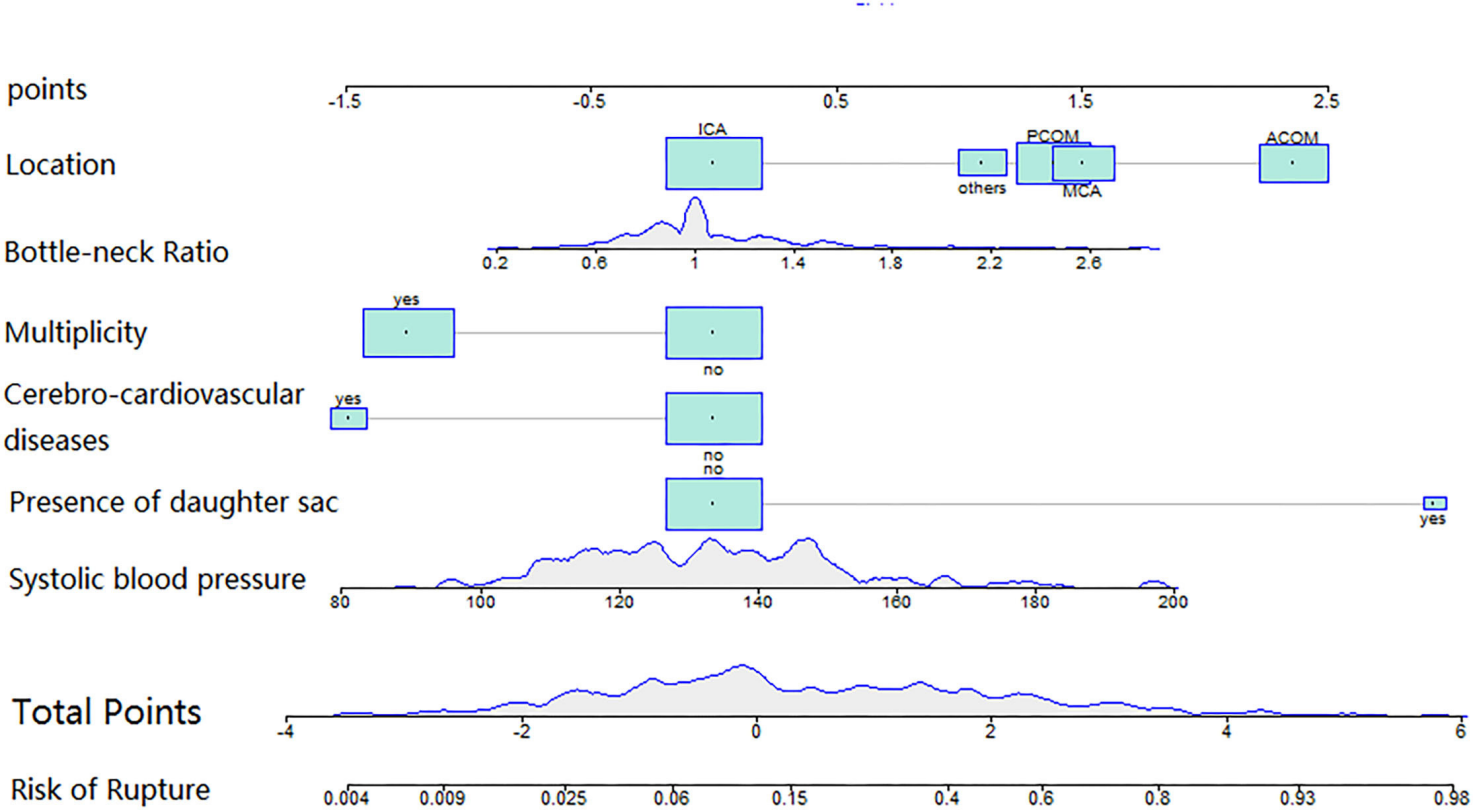
The nomogram was plotted based on 6 independent factors. The size of the bars indicates the number for each category, and the density plot represented the population distribution of each continuous variable. The total points, deriving from the sum points of the six individual indexes, determined the risk of aneurysm rupture.

A total of 343 patients with 427 small IAs were registered to the cohort. After excluding 46 aneurysms with unavailable morphological and demographic data, 381 aneurysms were included, of which 96 ruptured and 285 unruptured (Figure 1). The pre-treatment clinical and morphological characteristics and comparisons between groups were showed in Supplement Table 1. Multivariable analysis suggested that IA rupture was dependent on: higher systolic blood pressure (BP) at admission (*p* < 0.001, adjusted odds ratio [*OR*]: 1.029, 95% *CI*: 1.015–1.044), the presence of daughter sac (*p* < 0.001, adjusted *OR*: 19.042, 95% *CI*: 5.086–71.294), larger bottle-neck ratio (*p* = 0.007, adjusted *OR*: 2.801, 95% *CI*: 1.326–5.919), middle cerebral artery (MCA) (*p* = 0.002, adjusted *OR*: 4.526, 95% *CI*: 1.73–11.836), anterior communicating artery (Acom) (*p* < 0.001, adjusted *OR*: 10.601, 95% *CI*: 4.416–25.448), and posterior communicating artery (Pcom) (*p* = 0.002, adjusted *OR*: 3.997, 95% *CI*: 1.657–9.639) locate, while, cerebro-cardiovascular complications (*p* = 0.009, adjusted *OR*: 0.228, 95% *CI*: 0.075–1.044) and multiple aneurysms (*p* < 0.001, adjusted *OR*: 0.287, 95% *CI*: 0.153–0.538) were negative predictors (Supplement Table 2). Nomogram constructed based on these factors is shown in Figure 2.

**Figure 3:**
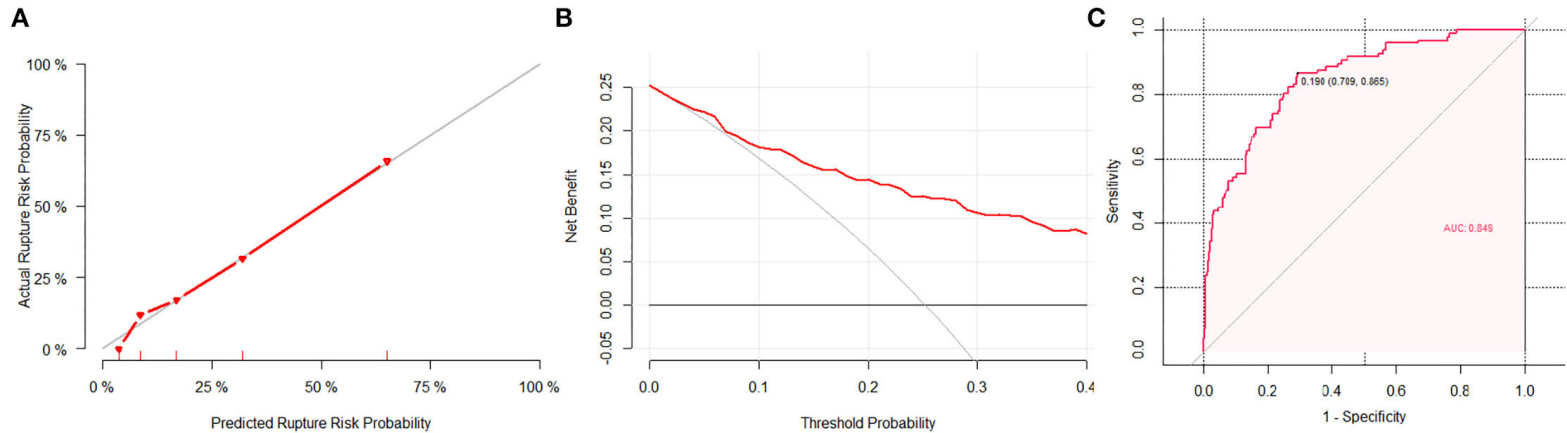
The performance of the nomogram. **(A)** A calibration plot showed the agreement between the average predicted risk of rupture (*x*-axis) and the observations (*y*-axis). The gray line represents the ideal calibration. The red line represents the actual performance of the nomogram, showing a close fit to the gray line and thus suggesting a good prediction. **(B)** In decision curve analysis, the horizontal axis is the threshold used to define high risk, the vertical axis is net benefit (NB). The red line represents the NB for the nomogram. The horizontal line at NB = 0 represents no intervention in the population (treat-none) and the gray curve shows the NB of taking intervention to everyone in the population regardless of rupture risk (treat-all). **(C)** The area under curve (AUC) of the nomogram was 0.849, with a sensitivity of 86.5% and a specificity of 70.9%.

The calibration plot showed good overall agreement between the predicted risk and observed events, as the reliability diagram almost overlapped with the diagonal line. The Hosmer–Lemeshow test yielded non-significant statistics (*p* = 0.82, X-square = 4.40), indicating a good model fitting. The DCA suggested that given any selected risk threshold, the nomogram achieved more benefit than both the treat-all policy and treat-none policy. The sensitivity, specificity, and accuracy of nomogram were 86.5%, 70.9%, and 74.7% in the observed data (Figure 3), respectively, with an optimal cut-off value of 0.19. While the PHASES score reached the sensitivity, specificity, and accuracy of 83.6%, 48.8%, and 57.5%, respectively, with an optimal cut-off value of 3. As compared with PHASES risk score (AUC = 0.693, 95% *CI* = 0.595–0.734), the nomogram model (AUC = 0.849, 95% *CI* = 0.805–0.893) had a better discriminatory ability (DeLong's test, *p* < 0.001). After applying the PHASES score to our subjects, the point for population was 3, and the point for size of aneurysm was 0. The PHASES risk score in our study ranged from 3 to 9.

## Discussion

In this study, we investigated risk factors and established a predictive nomogram for the risk of small IAs (max diameter < =5 mm) rupture in the eastern Asian population. Higher systolic BP at admission, no history of cardio-cerebrovascular complications, located at MCA, Acom, and Pcom, presence of daughter sac, presence of single aneurysm, and larger bottle-neck ratio were associated with a higher rupture risk. The performance of the nomogram was well demonstrated by the ROC, DCA and calibration plot.

Higher systolic BP at admission was identified as a significant risk factor. Since abrupt subarachnoid aneurysmal hemorrhage was usually associated with increased BP, the finding needs more investigations in prospective analyses to avoid bias from this retrospective study. Our results showed that patients with cardiovascular and cerebrovascular burden who were possibly given more attention on medical care and who received anti-inflammatory medication had a reduced chance of rupture. The presence of a daughter sac identified as significant risk factor in our study that has been widely acknowledged. Aneurysms located in Acom, Pcom, and MCA were found to have significantly higher rupture risk than those in other locations, which agreed with the previous studies (Suzuki et al., 2019). The larger aneurysm bottleneck factor increased ruptured risk, which shows similar consistency with other two studies (Hoh et al., 2007; Jiang et al., 2015). Actually, it has been claimed before that the geometry can affect the rupture of an IA by influencing the intra-aneurysmal hemodynamics. Aneurysms with larger bottle-neck ratio tend to have complex fluid dynamics that destabilize the aneurysmal structure (Ryu et al., 2011). Multiplicity seemed to be a trigger of small aneurysm rupture in Japanese (Sonobe, 2010), while we considered the uneven distribution of multiple aneurysms between cohorts in the study that may have led to a biased result. The reason why multiple aneurysms have a higher risk of rupture remains ambiguous. As Shi et al. (2021) found no significant association between aneurysmal multiplicity and rupture in the Chinese population, our study told the other story. Multiple aneurysms are more common in the older adults in this study cohort. Given that small aneurysms are more likely to rupture in younger patients (Suzuki et al., 2019), our finding of lower rupture risk in patients with multiple aneurysms seemed explicable, but the mechanism is still unclear and needs further exploration.

Various models and paradigms have been proposed to identify subjects at high risk of rupture. Such as the PHASES score, which was acknowledged as a generalized method of IA rupture risk evaluation. Despite its high diagnostic value, it was not specifically devised for the small ones, since distinctive pathophysiological presentations of small aneurysms are different from others (Kataoka et al., 1999). Several attempts have been made for IAs smaller than 7 mm. A Multi-View Convolutional Neural Network was established to assess the rupture risk of small IAs (<7 mm) (Ahn et al., 2021). Zhu et al. evaluated the growth and rupture risk of small IA (<7 mm) with explored risk factors (Zhu et al., 2021). For IAs smaller than 5 mm, Shi et al. developed a machine learning-based model with hemodynamic parameters for the rupture prediction of IAs. The AUC of their model reached 0.91 and 0.82 in the internal and external validation datasets, respectively (Shi et al., 2021). There is a wide range of potential approaches when developing a predictive model, each of which comes with trade-offs in accuracy and interpretability. Machine learning approaches perform well at the cost of interpretability, while we supposed that logistic regression tends to have slightly less accurate predictive performance but high interpretability. The use of nomogram has grown markedly recently, since it has been admitted as a reliable tool to quantify risk by incorporating important factors for oncologic prognoses. The advantages of a nomogram-based scoring schema are easy-to-use in clinical setting, and with points calculated in each category of indicator/risk factor, it is more understandable for clinicians when they need to make more comprehensive decisions on the identification and stratification of at-risk patients, based on a weighted scale or chart similar to a nomogram (Liang et al., 2015). In our study, the proposed nomogram incorporated easily accessible factors and achieved good performance (AUC = 0.85), showing a better performance than the PHASES score (AUC = 0.69). The nomogram may serve as a clinically easy-to-use decision supportive tool that facilitated individualized risk stratification and decision-making in managing patients with unruptured IAs smaller than 5 mm.

Our study had several limitations. First, this study was retrospective that may introduce a risk of bias. A prospective study design is required in the future. Second, the rupture of aneurysm itself might exert an influence on variables, such as systolic BP at admission and morphological features, accounting for another bias in our analysis. Third, the nature of the cross-sectional data is an imperfection of this study, and a longitudinal study design of follow-ups is required to explore the temporal relation between the rupture and the risk model. Furthermore, the clinical applicability of the model should be investigated in external datasets.

## Conclusion

This study developed a nomogram to predict rupture risk for IAs less than 5 mm. The constructed nomogram based on accessible clinical and morphological features shows a reasonably good predictive capability, and can be easily used by clinicians for individualized decision-making and management. Further studies designed in a multi-center setting and validated externally can be expected.

## Data Availability Statement

The raw data supporting the conclusions of this article will be made available by the authors, without undue reservation.

## Ethics Statement

The studies involving human participants were reviewed and approved by Research Ethics Committee of the First Affiliated Hospital, School of Medicine, Zhejiang University. The Ethics Committee waived the requirement of written informed consent for participation.

## Author Contributions

HL, JY, JP, and KN: conception and design of study. TZ, JW, WF, CG, MY, TC, and TZ: collection of data. KN, JP, and JM: analysis and/or interpretation of data. HL, KN, and JP: drafting the manuscript. JY, JP, RZ, and JM: revising the manuscript critically for important intellectual content. All authors contributed to the article and approved the submitted version.

## Funding

This research was funded by the Key Research & Development (R&D) Plan of Zhejiang Province (No. 2019C03034). The funder had no involvement in this study.

## Conflict of Interest

KN and JY were employed by Taimei Medical Technology. The remaining authors declare that the research was conducted in the absence of any commercial or financial relationships that could be construed as a potential conflict of interest.

## Publisher's Note

All claims expressed in this article are solely those of the authors and do not necessarily represent those of their affiliated organizations, or those of the publisher, the editors and the reviewers. Any product that may be evaluated in this article, or claim that may be made by its manufacturer, is not guaranteed or endorsed by the publisher.

## Abbreviations

IA: intracranial aneurysm
UIA: unruptured intracranial aneurysm
AUC: area under curve
OR: odds ratio
CI: confidence interval
INR: international normalized ratio
BP: blood pressure
DCA: decision curve analysis.
