# Supplementary material for: Nomogram-Based Risk Model of Small (≤5 mm) Intracranial Aneurysm Rupture in an Eastern Asian Study
Source: Front Aging Neurosci. 2022 May 11;14:872315. doi: 10.3389/fnagi.2022.872315 (PMC9132250; doi:10.3389/fnagi.2022.872315)
Supplement: Supplementary file 1 [file Data_Sheet_1.pdf]

Supplemental Tables

Table 1: Characteristics and comparisons between aneurysm cohorts.

	Total number of sIAs	Ruptured	Unruptured	P-value
Total	381	96	285	
Female	204 (53.5%)	44 (45.8%)	160 (56.1%)	0.08
Age	58.06±9.91	56.17±11.94	58.69±9.05	0.062
INR at admission	0.97±0.13	0.98±0.07	0.97±0.14	0.03*
Systolic BP at admission	133.56±19.94	141.26±22.19	130.97±18.46	<0.001*
Diastolic BP at admission	79.04±12.75	83.81±12.93	77.44±12.30	<0.001*
Antiplatelet and anticoagulant used	37 (9.7%)	3(3.1%)	34(11.9%)	0.012*
Presence of cerebro-cardiovascular complications	48 (12.6%)	5(5.2%)	43(15.1%)	0.012*
Hypertension	186(48.8%)	50(52.1%)	136(47.7%)	0.459
Diabetes	30(7.9%)	7(7.3%)	23(8.1%)	0.806
Hyperlipidemia	102(26.8%)	27(28.1%)	75(26.3%)	0.729
Family history of IA	2(0.5%)	1(1.0%)	1(0.4%)	0.441
Alcohol intake	53(13.9%)	18(18.8%)	35(12.3%)	0.113
Smoking or ex-smoking	70(18.4%)	23(24.0%)	47(16.5%)	0.102
Aneurysmal morphology				
Bifurcation	79(20.7%)	26(27.1%)	53(18.6%)	0.076
Presence of daughter sac	19(5.0%)	15(15.6%)	4(1.4%)	<0.001*
Multiplicity	178(46.7%)	26(27.1%)	152(53.3%)	<0.001*
Max diameter	3.28±0.95	3.35±0.93	3.25±0.96	0.309
Aneurysm neck width	2.61±0.89	2.37±0.78	2.70±0.91	0.003*
Aneurysm height	2.62±0.93	2.71±0.95	2.66±0.91	0.100
Aneurysm width	2.68±0.92	2.72±0.96	2.66±1.13	0.842
Bottle-neck ratio	1.08±0.37	1.21±0.43	1.03±0.34	<0.001*
Height-width ratio	1.02±0.31	1.07±0.36	1.00±0.29	0.223
Aspect ratio	1.08±0.45	1.24±0.47	1.02±0.43	<0.001*
Location				
ACA	16(4.2%)	3(3.1%)	13(4.6%)	<0.001*
Acom	72(18.9%)	39(40.6%)	33(11.6%)	
Pcom	82(21.5%)	23(24.0%)	59(20.7%)	
ICA	136(35.7%)	10(10.4%)	126(44.2%)	
MCA	57(15.0%)	15(15.6%)	42(14.7%)	
PCA	3(0.8%)	1(1.0%)	2(0.7%)	
Others	2(0.5%)	1(1.0%)	1(0.4%)	
BA-VA	13(3.4%)	4(4.2%)	9(3.2%)	

INR, international normalized ratio; BP, blood pressure; IA, intracranial aneurysm; ACA, anterior cerebral artery; Acom, anterior communicating artery; Pcom, posterior communicating artery; ICA, intracranial carotid artery; MCA, middle cerebral artery; PCA, posterior cerebral artery; BA-VA, basilar artery-vertebral artery.* represented

for significant level<0.05.

Table 2: Multivariate analysis of risk factors for sIA rupture.

Variables	β	aOR(95%CI)	P value
systolic BP at admission	0.029	1.029(1.015-1.044)	<0.001*
Location			
ICA			Reference
MCA	1.51	4.526(1.73-11.836)	0.002*
Acom	2.361	10.601(4.416-25.448)	<0.001*
Pcom	1.385	3.997(1.657-9.639)	0.002*
Multiplicity	-1.250	0.287(0.153-0.538)	<0.001*
bottle-neck ratio	1.030	2.801(1.326-5.919)	0.007*
presence of daughter sac	2.947	19.042(5.086-71.294)	<0.001*
cerebro-cardiovascular complications	-1.480	0.228(0.075-1.044)	0.009*

BP, blood pressure; ICA, intracranial carotid artery; MCA, middle cerebral artery; Acom, anterior communicating artery; Pcom, posterior communicating artery; aOR, adjusted odds ratio; β= regression coefficient. * represented for significant level<0.05.
